# A cross-sectional study applying the PRECEDE model to explore factors influencing epidemic prevention behaviors among preschool educators

**DOI:** 10.1186/s12889-024-20865-3

**Published:** 2024-12-18

**Authors:** Yi-Ying He, Wei-Hsiang Huang, Chen-Yin Tung

**Affiliations:** 1 Department of Health Promotion and Health Education, College of Education, National Taiwan Normal University, Taipei, Taiwan; 2 Department of Early Childhood Care and Education, University of Kang-Ning, Taipei, Taiwan; 3 Fire Department, New Taipei City Government, New Taipei, Taiwan

**Keywords:** PRECEDE model, Preschool educators, Epidemic prevention behaviors

## Abstract

**Background:**

This study investigates the epidemic prevention behaviors of preschool educators and the factors influencing these behaviors, applying the PRECEDE model as a framework for analysis.

**Methods:**

A cross-sectional survey was conducted among 190 preschool educators from public and private institutions in Taipei City and New Taipei City. A 64-item self-developed questionnaire was used to assess epidemic prevention behaviors and their determinants. The instrument’s reliability was supported by internal consistency (Cronbach’s α ranging from 0.85 to 0.92), while its validity was confirmed through expert review, item analysis, and confirmatory factor analysis (CFA). Statistical analyses included hierarchical regression to examine the influence of predisposing, reinforcing, and enabling factors on behavior.

**Results:**

The model explained 54% of the variance in epidemic prevention behaviors. Enabling factors had the strongest influence (β = 0.46, *p* < 0.001), followed by reinforcing factors (β = 0.15, *p* < 0.05) and predisposing attitudes (β = 0.14, *p* < 0.05). Background variables, such as age (β = 0.23, *p* < 0.001) and years of service, collectively explained 14% of the variance.

**Conclusion:**

The findings underscore the critical role of institutional support and professional training in enhancing epidemic prevention practices among preschool educators. Recommendations include integrating disease prevention training into professional development initiatives.

**Supplementary Information:**

The online version contains supplementary material available at 10.1186/s12889-024-20865-3.

## Background

In 2015, the United Nations introduced the 2030 Agenda for Sustainable Development, which encompasses 17 Sustainable Development Goals (SDGs). SDG 3 emphasizes combating epidemics such as AIDS, tuberculosis, malaria, and neglected tropical diseases, as well as preventing and treating hepatitis, water-borne diseases, and other infectious illnesses by 2030 [1, 2]. The COVID-19 pandemic, emerging in 2020, posed unprecedented challenges, disrupting global health progress, including in Taiwan where the COVID-19 incidence was reported at 0.062%. Taipei City had the highest rate (0.173%), followed by New Taipei City (0.161%) and Keelung City (0.083%) [3]. This pandemic highlighted the urgent need to strengthen infectious disease prevention measures.

Preschool environments, characterized by close interactions between educators and young children, pose significant transmission risks for infectious diseases [4]. Outbreaks in such settings can lead to serious health threats and operational disruptions. For instance, during enterovirus outbreaks in Taiwan, cluster infections in childcare facilities often led to school closures due to delayed isolation measures, impacting children’s education, family routines, and creating economic strain [5].

Preschool educators are pivotal in shaping young children’s health behaviors, as children often model behaviors observed in caregivers and educators [4]. Their adherence to preventive measures directly influences children’s compliance with public health protocols, emphasizing their role as key health behavior facilitators [6].

The PRECEDE-PROCEED model, developed by Green and Kreuter, provides a comprehensive framework for understanding health behaviors through the assessment of predisposing factors (beliefs, attitudes, and knowledge), reinforcing factors (social and institutional support), and enabling factors (structural and environmental conditions) [7]. Studies indicate that predisposing attitudes, such as perceived disease vulnerability, can motivate preventive actions, while reinforcing factors like institutional support sustain long-term adherence to preventive measures. Enabling factors, including resource access and professional training, are crucial for translating intentions into practical behaviors by reducing implementation barriers [8–10].

Given the complexity and focus of this study, the PRECEDE model was applied to diagnose and assess these health behavior determinants without engaging in the implementation and evaluation phases characterized by the PROCEED component. Concentrating on predisposing, reinforcing, and enabling factors allows for identifying key influences on epidemic prevention behaviors, forming a foundation for future targeted interventions. The PRECEDE model has been effectively employed to understand and promote preventive behaviors in public health contexts, including early childhood education settings, enhancing health-promoting practices through tailored interventions and supportive policies [11–13]. In epidemic prevention, it provides a systematic assessment framework for educators’ needs, supporting the development of tailored strategies that address specific challenges and strengths.

This study aims to apply the PRECEDE model to analyze factors influencing epidemic prevention behaviors among preschool educators. By identifying key determinants, the study offers practical recommendations for educational and public health authorities and establishes a framework for future interventions that enhance epidemic prevention within this essential community.

## Method

### Research design and framework

This cross-sectional study employed a survey research design to investigate factors influencing epidemic prevention behaviors among preschool educators. The independent variables included participants’ background characteristics, epidemic prevention knowledge, attitudes (predisposing factors), reinforcing factors, and enabling factors, while the dependent variable was epidemic prevention behaviors. Background variables were treated as categorical, and epidemic prevention knowledge was measured using a binary true/false format. All other variables were assessed using multi-item Likert-scale questions.

Predisposing factors referred to the educators’ attitudes, beliefs, and motivations towards epidemic prevention. Reinforcing factors encompassed social and institutional support, such as encouragement from parents, colleagues, and policies. Enabling factors involved structural and resource-based supports, like institutional policies and training opportunities. Each of these constructs was measured using items rated on a 5-point Likert scale, where higher scores indicated stronger perceived support, agreement, or frequency of behaviors.

### Study participants and sample size estimation

According to the 2022 Ministry of Education statistics, the total number of preschool educators in New Taipei City was 9,229, comprising 2,639 in public preschools and 6,590 in private preschools. In Taipei City, there were 5,948 preschool educators, including 1,787 in public preschools and 4,161 in private preschools [14]. The overall population ratio between New Taipei City and Taipei City was approximately 5:3.

Participants for this study were recruited using convenience sampling from public and private preschools in New Taipei City and Taipei City between December 11 and December 15, 2023. A total of 250 preschool educators were initially recruited, including 60 participants for the pilot study, whose data were subsequently excluded from the main analysis. The final sample for the main study comprised 190 participants, with 136 from New Taipei City and 54 from Taipei City, maintaining a 5:2 ratio. Although this distribution does not precisely reflect the actual population proportions, it closely aligns with the general regional distribution and reflects the characteristics inherent to convenience sampling, which should be taken into account when interpreting the study’s findings.

### Research instrument

The research instrument used in this study was a self-developed questionnaire specifically designed to assess epidemic prevention behaviors and their influencing factors among preschool educators. A comprehensive review of existing domestic and international questionnaires highlighted the absence of a tool fully addressing the unique characteristics relevant to this population. International instruments posed potential challenges, such as cultural and linguistic differences, that could undermine validity and applicability. Consequently, after extensive discussions with experts and scholars, a self-developed questionnaire grounded in the PRECEDE model was deemed most suitable due to its established theoretical structure and proven effectiveness in evaluating health behaviors, as supported by numerous studies.

The initial development involved creating an 80-item instrument based on a thorough literature review, alignment with governmental health authority guidelines, and consultations with field experts to ensure content validity [15–17]. The instrument underwent expert validation, difficulty analysis, discrimination analysis, and item analysis. Items with a difficulty index below 0.3 or above 0.7 were removed [18], as well as those with a discrimination index below 0.2 [19], leading to the elimination of 9 items. Item analysis was further performed using an extreme group test, where items with critical ratios less than 3 or lacking significant differences were excluded [20], resulting in the removal of an additional 7 items. This refinement process led to a final version comprising 64 items. For detailed information on the item analysis process, scoring methods, difficulty indices, and reliability measures, please refer to Table 1.

**Table 1:** Structured questionnaire framework (*N* = 60)

Construct	Original Items	After Deletion	Scoring method	Overall Scoring	#Difficulty Analysis #Discrimination Analysis @Item Analysis	Reliability
Background	6	6	Category			
Predisposing Knowledge	19	10	1 or 0 points for correct and incorrect answers, respectively	0–10	#P: 0.15 − 1.00 #D: 0.03–0.31	KR-20:0.51
Predisposing Attitudes	14	9	5-point Likert scale	9–45	@cr:1.46–4.99*** @r:0.42**−0.69**	α: 0.85
Reinforcing	11	11	5-point Likert scale	11–55	@cr: 4.39***−6.79*** @r: 0.47***−0.75***	α: 0.89
Enabling	11	10	5-point Likert scale	10–50	@cr: 1.94–4.04*** @r: 0.56***−0.79***	α: 0.92
Prevention Behaviors	19	18	5-point Likert scale	18–90	@cr: 1.96–6.22*** @r: 0.55***−0.73***	α: 0.92

*P* Difficulty Index, *D* Discrimination Index, *cr* Critical Ratio, *r* Correlation Coefficient

**P<0.01, ***P<0.001

Construct validity was assessed through confirmatory factor analysis (CFA), focusing on convergent and discriminant validity. Convergent validity was verified via factor loadings, Average Variance Extracted (AVE ≥ 0.5), and Composite Reliability (CR ≥ 0.7) [21]. As shown in Table 2, the factor loadings for all constructs were above the recommended threshold, with CR values indicating strong reliability across the constructs. Discriminant validity was established by comparing AVE values with squared correlations among constructs, as detailed in Table 3. As our focus was on measurement model validation, fit indices for overall model evaluation were not included. Reliability was assessed through internal consistency measures using KR-20 and Cronbach’s α. The KR-20 value for predisposing knowledge (19 items) was 0.51, which, while indicating moderate reliability, may reflect the inherent challenges of binary-response items and variations in item difficulty [22]. Cronbach’s α values were 0.85 for predisposing attitudes (14 items), 0.89 for reinforcing factors (11 items), 0.92 for enabling factors (11 items), and 0.92 for epidemic prevention behaviors (19 items), aligning with established reliability standards [23]. This indicates strong internal consistency across these constructs.

**Table 2:** Summary of convergent validity from confirmatory factor analysis of the questionnaire (*N* = 190)

Construct	Items	SFL	SMC	CR	AVE
Predisposing Attitudes	N17	0.54	0.29	0.87	0.42
	N18	0.42	0.18		
	N19	0.39	0.15		
	N20	0.71	0.50		
	N21	0.75	0.56		
	N22	0.71	0.50		
	N23	0.66	0.46		
	N24	0.78	0.61		
	N25	0.71	0.50		
Reinforcing	N26	0.80	0.64	0.93	0.49
	N27	0.83	0.69		
	N28	0.79	0.62		
	N29	0.87	0.76		
	N30	0.58	0.33		
	N31	0.50	0.25		
	N32	0.49	0.24		
	N33	0.62	0.38		
	N34	0.56	0.31		
	N35	0.64	0.41		
	N36	0.60	0.36		
Enabling	N37	0.75	0.56	0.94	0.55
	N38	0.74	0.55		
	N39	0.82	0.67		
	N40	0.77	0.59		
	N41	0.78	0.61		
	N42	0.70	0.49		
	N43	0.77	0.59		
	N44	0.59	0.35		
	N45	0.76	0.58		
	N46	0.63	0.40		
Prevention Behaviors	N47	0.65	0.42	0.97	0.44
	N48	0.68	0.46		
	N49	0.69	0.48		
	N50	0.70	0.49		
	N51	0.65	0.42		
	N52	0.70	0.49		
	N53	0.63	0.40		
	N54	0.61	0.37		
	N55	0.59	0.35		
	N56	0.47	0.22		
	N57	0.67	0.45		
	N58	0.63	0.40		
	N59	0.64	0.41		
	N60	0.73	0.53		
	N61	0.71	0.50		
	N62	0.62	0.38		
	N63	0.60	0.36		
	N64	0.56	0.31		

*SFL* Standardized factor loading, *SMC* Square multiple correlation, *CR* Composite Reliability, *AVE* Average Variance Extracted

**Table 3:** Summary of discriminant validity from confirmatory factor analysis of the questionnaire (*N* = 190)

Construct	1	2	3	4
Prevention Behaviors	0.64			
Predisposing Attitudes	0.36***	0.65		
Reinforcing	0.47***	0.43***	0.67	
Enabling	0.63***	0.37***	0.63***	0.73

The values along the diagonal (in the first cell of each row and column intersection) represent the square root of the average variance extracted (AVE) for each construct. These values should be greater than the off-diagonal correlation values in their respective rows and columns

***p < 0.001

In summary, the final questionnaire comprised 64 items, including six background variables. The knowledge component was measured using a binary true/false format (correct, incorrect, don’t know), while the other constructs were assessed using a 5-point Likert scale. Responses for attitudes, reinforcing factors, and enabling factors ranged from “strongly agree” to “strongly disagree” (5 to 1), and epidemic prevention behaviors were measured from “always” to “never” (5 to 1), with higher scores indicating stronger agreement or more frequent behaviors. The English version of the questionnaire, titled “Survey on Current Epidemic Prevention Practices and Determinants Among Early Childhood Educators,” is provided as a supplementary file for reference.

### Statistical methods

Data analysis for this study was conducted using a combination of IBM SPSS version 22.0 for Windows, STATA version 14.0, and Microsoft Excel 2021. The following outlines the use of each software:

SPSS: Descriptive statistics, including frequency distributions, percentages, means, and standard deviations, were used to describe participant characteristics. Pearson correlation analysis examined relationships between continuous variables, while independent sample t-tests compared binary background variables. For categorical variables with more than two levels, one-way analysis of variance (ANOVA) was performed. Hierarchical regression analysis was used to evaluate the explanatory power of predisposing attitudes, reinforcing factors, enabling factors, and background variables in predicting epidemic prevention behaviors. Internal consistency for continuous and binary items was assessed using Cronbach’s α and KR-20, respectively.

STATA: Confirmatory factor analysis (CFA) was conducted to evaluate the convergent and discriminant validity of the measurement model. The CFA focused on factor loadings, Average Variance Extracted (AVE), and Composite Reliability (CR) to ensure robust construct measurement and validity.

Excel: Difficulty and discrimination analyses were performed using Microsoft Excel 2021. Item analysis for discrimination employed extreme group testing, removing items based on t-values less than 3 or those that did not achieve statistical significance.

All statistical tests were conducted at a significance level of α = 0.05, with a target power of 0.8 and a medium effect size to ensure adequate sensitivity in detecting meaningful relationships within the data.

## Results

### Background information of preschool educators

The analysis of participants’ background characteristics revealed that the majority worked in private preschools (*n* = 144, 75.8%), followed by public preschools (*n* = 32, 16.8%) and nonprofit preschools (*n* = 14, 7.4%) out of a total of 190 participants. Regarding age distribution, 39 participants (20.5%) were 25 years old or younger. Most participants held a university or technical college degree (*n* = 165, 86.8%). The largest job role group was caregivers (*n* = 107, 56.3%). Additionally, the highest proportion of educators had more than seven years of service (*n* = 76, 40.0%).

### Response patterns for predisposing knowledge, attitudes, reinforcing factors, enabling factors, and epidemic prevention behaviors

The analysis, as shown in Table 4, revealed that for predisposing knowledge (10 items), the mean score was 7.22 (SD = 1.56), indicating moderate to high levels of knowledge among preschool educators. Among these responses, a “don’t know” option was selected by participants on specific items, with percentages ranging from 0.5 to 7.9% across the questions. This suggests areas where knowledge gaps may exist, potentially impacting the educators’ confidence or accuracy in implementing epidemic prevention measures.

**Table 4:** Summary of questionnaire response analysis (*N* = 190)

Construct	Brief Item Descriptions	Total no. of items	Overall mean score	Overall SD	Mean among items	SD among items	Correct Responses (%)	“Don’t Know” Responses (%)
Predisposing Knowledge	N7 Maintain 1-meter indoor social distance unless masked.	10	7.22	1.56			23.16	1.58
	N8 Wash hands with soap for 10–15 s.						88.42	0.53
	N9 Avoid face-touching during enterovirus outbreaks.						92.63	1.05
	N10 Handwash after outdoor activities to prevent germs.						73.16	2.11
	N11 Cover sandpits to prevent contamination and salmonella risk.						86.84	7.89
	N12 Dengue is transmitted by mosquitoes, not person-to-person.						67.37	7.37
	N13 Chickenpox affects infants under one year.						87.89	3.68
	N14 Vaccination prevents chickenpox in ~ 90% of cases.						80.53	6.32
	N15 Disinfect surfaces with bleach during COVID-19.						25.79	0.53
	N16 Cook food thoroughly; avoid raw food and shared utensils.						96.32	0.53
Predisposing Attitudes	N17 Educators remind parents/children about flu vaccination.	9	40.16	5.26	4.63	0.74		
	N18 Concern over legal responsibilities for prevention non-compliance.				4.02	1.05		
	N19 Worry about lacking knowledge for effective prevention.				4.18	1.09		
	N20 Confidence in increasing handwashing to prevent disease.				4.54	0.81		
	N21 Confidence in following proper handwashing steps.				4.61	0.76		
	N22 Confidence in seeking medical attention for symptoms.				4.57	0.75		
	N23 Confidence in wearing masks in crowded/public transport.				4.62	0.79		
	N24 Confidence in disinfecting surfaces with bleach/alcohol.				4.44	0.97		
	N25 Staying informed through news/social media.				4.56	0.81		
Reinforcing	N26 Parents inform preschool about child’s health/absence.	11	46.64	7.39	4.17	1.00		
	N27 Parents keep sick children home to prevent spread.				4.03	1.09		
	N28 Parents prepare masks/reminders during flu season.				4.38	0.75		
	N29 Parents take sick children to the doctor and follow prevention.				4.32	0.87		
	N30 Preschools monitor educator health, nutrition, and rest.				4.36	0.88		
	N31 Preschools offer vaccination incentives.				4.08	1.13		
	N32 Preschools encourage disease prevention training.				3.84	1.32		
	N33 Preschools promote staying home when sick.				4.15	1.17		
	N34 In-service health training supports prevention adoption.				4.39	0.81		
	N35 Ministry promotes disease prevention via media.				4.44	0.80		
	N36 Education bureaus enforce disease control measures.				4.48	0.73		
Enabling	N37 Preschools provide masks, gloves, and disinfectants.	10	46.26	4.79	4.55	0.73		
	N38 Preschools ensure safe drinking water through testing.				4.73	0.63		
	N39 Preschools maintain clean kitchens for food safety.				4.77	0.48		
	N40 Preschools remind staff about flu vaccines.				4.67	0.58		
	N41 Preschools update staff on infectious diseases.				4.62	0.60		
	N42 Handwashing stations installed at preschool.				4.64	0.65		
	N43 Hand disinfection required before entry.				4.69	0.57		
	N44 Sick child policy helps monitor attendance.				4.54	0.69		
	N45 Disease contingency plan for outbreaks.				4.58	0.63		
	N46 Enterovirus reporting drills aid monitoring.				4.48	0.77		
Prevention Behaviors	N47 Monitor current disease situation.	18	80.55	9.1	4.27	0.83		
	N48 Provide disease prevention info at preschool.				4.12	0.95		
	N49 Learn/follow disease prevention guidelines.				4.51	0.62		
	N50 Check children’s health upon arrival; enforce sick child stay-home policy.				4.43	0.74		
	N51 Monitor sick leave and enforce prevention measures.				4.58	0.68		
	N52 Study disease prevention and create health lessons.				4.34	0.84		
	N53 Practice hand hygiene during school interactions.				4.65	0.64		
	N54 Teach children proper cough/sneeze etiquette.				4.71	0.57		
	N55 Wear masks when sick and follow respiratory hygiene.				4.76	0.48		
	N56 Get flu and COVID-19 vaccines.				4.40	0.94		
	N57 Maintain diet, exercise, and sleep for immunity.				4.21	0.93		
	N58 Stay home when sick to avoid spreading illness.				4.35	0.90		
	N59 Ensure good ventilation and cleanliness at preschool.				4.64	0.65		
	N60 Learn and adhere to prevention measures.				4.60	0.67		
	N61 Share flu-related info in communication books during flu season.				4.42	0.89		
	N62 Wear masks in crowded places.				4.68	0.61		
	N63 Clean frequently touched items (phone, keyboard).				4.33	1.01		
	N64 Seek medical attention for symptoms like sore throat/fever.				4.56	0.74		

For predisposing attitudes (9 items), the mean score was 40.16 (SD = 5.26), reflecting generally positive attitudes toward managing infectious disease outbreaks. Reinforcing factors (11 items) had a mean score of 46.64 (SD = 7.39), showing that educators valued support from parents, institutions, and government policies. The mean score for enabling factors (10 items) was 46.26 (SD = 4.79), highlighting the perceived importance of institutional support and a preventive environment. Epidemic prevention behaviors (18 items) had a mean score of 80.55 (SD = 9.1), indicating high levels of implementation during outbreaks.

### Differences and correlations among variables

No significant differences in epidemic prevention behaviors were observed between educators from New Taipei City and Taipei City (t(188) = 0.63, *p* = 0.53, d = 0.10). However, significant positive correlations were identified between epidemic prevention behaviors and predisposing attitudes (γ = 0.36, *p* < 0.001), reinforcing factors (γ = 0.47, *p* < 0.001), and enabling factors (γ = 0.63, *p* < 0.001). There were no significant differences by preschool type (F(189) = 1.08, *p* = 0.34, η² = 0.04). However, age (F = 3.36, *p* < 0.01, η² = 0.10) and years of service (F = 4.93, *p* < 0.01, η² = 0.07) showed significant, albeit small, effects. Scheffé post hoc analysis indicated that educators aged 51 and older (M = 88.17, SD = 3.66) exhibited stronger epidemic prevention behaviors compared to those aged 26–30 (M = 74.57, SD = 10.08). Similarly, educators with more than seven years of service (M = 83.24, SD = 8.38) outperformed those with 4–6 years of service (M = 76.87, SD = 7.79).

### Explanatory power of variables

Using the PRECEDE framework and previous research [11, 24], four hierarchical regression models were developed to examine the explanatory power of predisposing attitudes, reinforcing factors, and enabling factors on epidemic prevention behaviors after controlling for background variables (Table 5).

**Table 5:** Summary of hierarchical regression analysis of the questionnaire (*N* = 190)

Variables	Model 1		Model 2			Model 3		Model 4		
	Background factors		Background and predisposing attitudes factors			Background, predisposing attitudes, and reinforcing factors		Background, predisposing attitudes, reinforcing and enabling factors		Collinearity diagnostic
	β	t	β		t	β	t	β	t	VIF
Background variables										
Ages 26–30	−0.10	−1.29	−0.16	−2.23*		−0.16	−2.41*	−0.12	−2.04*	1.37
Ages 31–35	0.08	0.77	−0.02	−0.25		−0.03	−0.35	0.01	0.02	2.08
Ages 36–40	0.08	0.73	0.03	0.27		0.05	0.60	0.08	1.00	2.20
Ages 41–45	0.12	1.25	0.07	0.80		0.11	1.38	0.14	1.92	1.89
Ages 46–50	0.10	1.04	0.03	0.37		0.03	0.34	0.06	0.87	1.90
Ages 51 and above	0.25	2.94**	0.21	2.67**		0.21	2.99**	0.23	3.68***	1.50
Years of work experience 1–3 years	0.03	0.30	0.04	0.53		0.11	1.51	0.08	1.23	1.60
Years of work experience 4–6 years	−0.16	−1.73	−0.13	−1.55		−0.05	−0.61	− 0.08	−1.18	1.92
Years of work experience 7 years and above	0.09	0.80	0.11	1.00		0.21	2.00*	0.07	0.80	2.94
Predisposing attitudes factors			0.38	5.70***		0.21	3.12**	0.14	2.31*	1.33
Reinforcing factors						0.45	6.59***	0.15	2.17*	2.03
Enabling factors								0.46	6.65***	1.87
R^2^	0.14		0.28			0.42		0.54		
Adj R^2^	0.10		0.24			0.38		0.50		
F	3.33***		6.84***			11.64***		16.95***		
△R^2^	0.14		0.13			0.14		0.12		
△F	3.33**		16.53***			46.60***		41.25***		

*VIF* Variance inflation factor

*p < 0.05

**p < 0.01

***p < 0.001

### Model 1: background factors

In Model 1, background factors such as age and years of service were included. This model explained 14% of the variance in epidemic prevention behaviors (adjusted R² = 0.10, F = 3.33, *p* < 0.001), indicating that both age and years of service significantly contributed to the variance explained.

### Model 2: adding predisposing attitudes

When predisposing attitudes were added in Model 2, the explained variance increased to 28% (adjusted R² = 0.24, ΔR² = 0.13, F = 6.84, *p* < 0.001). Predisposing attitudes had a positive and significant effect on epidemic prevention behaviors, contributing an additional 13% of explained variance (β = 0.14, t = 2.31, *p* < 0.05).

### Model 3: adding reinforcing factors

Model 3 included reinforcing factors, increasing the explained variance to 42% (adjusted R² = 0.38, ΔR² = 0.14, F = 11.64, *p* < 0.001). The addition of reinforcing factors provided a significant improvement in explaining epidemic prevention behaviors, with a β coefficient of 0.15 (t = 2.17, *p* < 0.05).

### Model 4: adding enabling factors

In Model 4, enabling factors were introduced, further increasing the explained variance to 54% (adjusted R² = 0.50, ΔR² = 0.12, F = 16.95, *p* < 0.001). Enabling factors emerged as the strongest predictor of epidemic prevention behaviors (β = 0.46, t = 6.65, *p* < 0.001). Age (specifically, educators aged 51 and older) also remained a significant contributor (β = 0.23, t = 3.68, *p* < 0.001).

Overall, the final model demonstrated that background factors (age), predisposing attitudes, reinforcing factors, and enabling factors together provided the greatest explanatory power for epidemic prevention behaviors, with no multicollinearity detected (VIF < 10).

## Discussion

This study applied the PRECEDE model to examine the factors influencing epidemic prevention behaviors among preschool educators. Previous research has consistently shown a significant positive relationship between preventive behaviors for infectious diseases and individuals’ health beliefs and attitudes [11, 25, 26]. While many studies have employed the Health Belief Model and the Theory of Planned Behavior to explore health behavior determinants, fewer have investigated the hierarchical strength of these influences across variables. By focusing on the predisposing phase of the PRECEDE model, this study provides new insights into the determinants of epidemic prevention behaviors, offering practical implications for the development of intervention measures and policies aimed at improving these behaviors among preschool educators.

### Differences in epidemic prevention behaviors by background variables

The results indicated significant differences in epidemic prevention behaviors based on age and years of service. Educators aged 51 and above demonstrated significantly stronger epidemic prevention behaviors compared to those aged 26–30. Similarly, educators with more than seven years of service exhibited stronger behaviors than those with 4–6 years of service.

These findings are consistent with earlier studies suggesting that older individuals are more likely to engage in effective preventive behaviors. For instance, a study involving 2,256 adults found that older adults exhibited stronger associations with perceived behavioral control (PBC) across a range of preventive behaviors compared to younger adults [25]. Similarly, a study of 380 Egyptian adults during the COVID-19 pandemic, utilizing the Health Belief Model, found that older age and higher education levels were associated with better preventive behaviors [26]. These results underscore the potential impact of age and experience on preventive practices.

### Correlation between predisposing attitudes, reinforcing factors, enabling factors, and epidemic prevention behaviors

This study revealed significant positive correlations between predisposing attitudes, reinforcing factors, enabling factors, and epidemic prevention behaviors. However, no significant correlation was found between predisposing knowledge and epidemic prevention behaviors.

These findings align with previous research. For example, a study of 240 medical students during the COVID-19 pandemic found no statistically significant relationship between COVID-19 knowledge and preventive behaviors. However, a significant negative correlation was observed between preventive behaviors and risk perception, indicating that as preventive behaviors increased, perceived risk decreased [27]. While the positive correlations between attitudes and behaviors observed in our study highlight important associations, they should be interpreted as non-causal. Adequate protective equipment and training have been shown to build confidence in managing epidemics, which, in turn, supports proactive preventive actions. Similarly, a study of 3,190 Turkish adults found that COVID-19 knowledge alone was not a significant predictor of preventive behaviors [28].

### Explanatory power of predisposing attitudes, reinforcing factors, and enabling factors

The PRECEDE model posits that sustained health behaviors are influenced by a combination of predisposing, reinforcing, and enabling factors. In this study, older age and longer years of service were associated with stronger epidemic prevention behaviors. Positive predisposing attitudes were linked to increased motivation for health-promoting behaviors, which in turn served as reinforcing factors. Social support from parents, preschools, and government policies further enhanced these reinforcing factors, transforming them into enabling factors. Institutional support, such as preventive environments and training programs, played a crucial role in shaping the epidemic prevention behaviors of preschool educators.

The findings are consistent with prior applications of the PRECEDE model, which have demonstrated its utility in improving health behaviors through the enhancement of predisposing, reinforcing, and enabling factors. For example, interventions using this model to prevent otitis media in children emphasized the importance of family and healthcare support, as well as access to educational materials, to improve preventive behaviors [29]. In the context of preschools, educators’ positive attitudes toward infectious disease prevention have been closely linked with more frequent implementation of preventive behaviors, supported by social and institutional backing [17, 30]. Established infection control systems within preschools further underscore the importance of supportive environments in promoting effective preventive behaviors [11, 31].

## Conclusions

### Key associations identified

The study found that beyond background characteristics such as age and years of service, predisposing attitudes, reinforcing factors, and enabling factors were significantly associated with epidemic prevention behaviors among preschool educators.

### Enabling factors as strongest predictors

Enabling factors demonstrated the strongest positive association (β = 0.46), underscoring the importance of institutional support and resources.

### Explained variance

Background characteristics, predisposing attitudes, reinforcing factors, and enabling factors collectively accounted for 54% of the variance in epidemic prevention behaviors, contributing 14%, 13%, 14%, and 12%, respectively.

### Significance of age

Educators aged 51 and older (β = 0.23) displayed significantly higher engagement in prevention behaviors compared to younger educators, highlighting the association between age, experience, and prevention behaviors.

### Practical implications

These findings highlight the necessity of creating supportive environments within preschools, ensuring that educators have access to tools, training, and institutional backing needed to support effective epidemic prevention behaviors. 

### Suggestions

#### Recommendations for preschool practice

Given that educators aged 51 and above exhibited stronger epidemic prevention behaviors, preschools should consider leveraging their experience by integrating these individuals into leadership or advisory roles. This approach fosters a culture of preparedness and prevention, aligns with policies promoting the re-employment of older adults, and enhances institutional resilience in managing health crises.

Additionally, preschools are encouraged to establish comprehensive business continuity planning (BCP) to ensure timely and effective epidemic prevention measures, minimize operational disruptions, and safeguard the health of staff and students.

#### Recommendations for educational authorities

To further strengthen epidemic prevention behaviors, government agencies should consider revising the Regulations for the Implementation of Professional Knowledge Training for Preschool Educators (Article 2) to integrate infectious disease prevention into ongoing education. Additionally, revising emergency preparedness and response protocols could enhance educators’ readiness and response capabilities during health crises, ensuring effective implementation of preventive behaviors.

#### Recommendations for future research

##### Establishing causal links

Employ experimental or longitudinal designs to establish clearer causal relationships between variables such as enabling factors and epidemic prevention behaviors.

##### Exploring risk perception

Investigate the impact of epidemic prevention behaviors on risk perception among preschool educators to better understand how perceived risks influence behaviors.

##### Involving preschool students

Study the role of preschool students in epidemic prevention efforts and explore the interaction between educators’ and students’ preventive behaviors to foster a school-wide culture of epidemic prevention.

##### Incorporating diverse contexts

Expand research to include diverse geographic and professional contexts to enhance the generalizability of findings and explore how context-specific factors may influence epidemic prevention behaviors.

##### Refining measurement scales

Future studies should consider refining item construction and increasing item diversity to enhance scale reliability. This includes ensuring a balanced distribution of item difficulty and improving discrimination power, particularly for binary-response scales, to address limitations observed in current reliability measures.

##### Integrating mixed methods

Utilize a combination of quantitative and qualitative approaches, such as interviews and focus groups, to gain deeper insights into the interpretation and application of epidemic prevention behaviors by preschool educators. This could inform more comprehensive and effective intervention designs. 

### Limitations

#### Internal validity

##### Complexity of the PRECEDE-PROCEED model

While effective, the complexity and rigidity of the model may limit its adaptability in certain contexts. Future research could explore the integration of alternative theories such as the Theory of Planned Behavior or Social Cognitive Theory.

##### Potential response bias

The use of a self-administered questionnaire may introduce potential biases such as non-response bias and social desirability bias. Although these limitations exist, the researchers have taken several measures to minimize their impact, including:

- Anonymity Assurance: Ensuring respondent anonymity to encourage honest and accurate responses.
- Pilot Testing: Conducting a pilot study to refine questionnaire items for clarity and comprehensiveness, aiming to reduce potential response biases.
- Questionnaire Design: Utilizing neutral wording and diverse response formats to mitigate leading questions and minimize social desirability influences. Despite these efforts, some level of bias may still be present and is acknowledged as a limitation of this study.

 

##### Impact of item quantity and difficulty

The relatively lower KR-20 value observed for the predisposing knowledge scale reflects the inherent limitations of binary-response items, including restricted variance compared to continuous measures and greater sensitivity to item difficulty and discrimination. Binary items offer fewer response options, which can lead to lower variability and, subsequently, reduced reliability estimates. Additionally, the limited measurement precision and potential skew in response distributions further contribute to this phenomenon.

#### External validity

##### Geographic scope

The study focused solely on preschool educators from Taipei City and New Taipei City, limiting the generalizability of the findings to other regions or professional groups.

##### Sample diversity

The limited demographic and professional scope may not fully represent the broader population of preschool educators. Future studies should aim to include a more diverse and representative sample to enhance external validity.

## Supplementary Information

Supplementary Material 1.

## Data Availability

The datasets generated and/or analyzed during the current study are available from the corresponding author upon reasonable request.

## Abbreviations

SDGs: Sustainable Development Goals
COVID-19: Coronavirus Disease 2019
PPM: PRECEDE-PROCEED Model
CFA: Confirmatory Factor Analysis
KR-20: Kuder-Richardson Formula 20
BCP: Business Continuity Planning
PBC: Perceived Behavioral Control
ANOVA: Analysis of Variance
VIF: Variance Inflation Factor
