# Supplementary material for: A cross-sectional study applying the PRECEDE model to explore factors influencing epidemic prevention behaviors among preschool educators
Source: BMC Public Health. 2024 Dec 18;24:3486. doi: 10.1186/s12889-024-20865-3 (PMC11657516; doi:10.1186/s12889-024-20865-3)
Supplement: Supplementary file 1 — Supplementary Material 1. [file 12889_2024_20865_MOESM1_ESM.docx]

Survey on Current Epidemic Prevention Practices and Determinants Among Early Childhood Educators

Please answer the following questions based on your actual situation. Mark "✓" on the appropriate option.

# Part 1: Basic Information

1. Area of preschool: (1) □ New Taipei City (2) □ Taipei City

2. Nature of preschool: (1) □ Private (2) □ Public (3) □ Non-profit

3. Age: (1) □ 25 or below (2) □ 26-30 (3) □ 31-35 (4) □ 36-40 (5) □ 41-45 (6) □ 46-50 (7) □ 51 or above

4. Education: (1) □ High School or below (2) □ College (3) □ Graduate

5. Current position: (1) □ Principal (2) □ Teacher (3) □ Caregiver (4) □ Assistant Caregiver

6. Years of service: (1) □ Less than 1 year (2) □ 1-3 years (3) □ 4-6 years (4) □ 7 years or more

# Part 2: Predisposing Factors - Knowledge

Please choose the correct answer for each statement.

|  | Correct | Incorrect | Don’t Know |
| --- | --- | --- | --- |
| N7. During the pandemic, maintain a 1-meter indoor social distance, unless wearing a mask correctly. |  |  |  |
| N8 Wash hands with soap or disinfectant for at least 10-15 seconds each time. |  |  |  |
| N9 During an enterovirus outbreak, remind children to avoid touching their face to reduce infection risk from contaminated items or contact with infected children. |  |  |  |
| N10 Handwashing before entering the preschool after outdoor activities prevents the spread of microorganisms. |  |  |  |
| N11 Cover outdoor sandpits when not in use to prevent contamination from animal feces and reduce the risk of salmonella. |  |  |  |
| N12 Dengue fever is caused by the dengue virus, transmitted by mosquitoes, not from person to person or via air/contact. |  |  |  |
| N13 Chickenpox, caused by the varicella virus, affects only infants under one year old. |  |  |  |
| N14 Vaccination can prevent chickenpox, with about 90% of recipients developing immunity. |  |  |  |
| N15 During COVID-19, disinfect household surfaces like doorknobs and tables with bleach. Higher bleach concentration improves effectiveness. |  |  |  |
| N16 Cook food thoroughly, avoid raw foods, and use shared utensils to reduce the risk of bacterial intestinal infections. |  |  |  |

# Part 3: Predisposing Factors - Attitudes

Please indicate your level of agreement with the following statements.

|  | Strongly Disagree | Disagree | Neutral | Agree | Strongly Agree |
| --- | --- | --- | --- | --- | --- |
| N17 I believe preschool educators should use various methods (e.g., communication books, verbal reminders) to remind parents and children of the importance of flu vaccination in preventing infectious diseases. |  |  |  |  |  |
| N18 I am concerned about the legal responsibilities of not following infectious disease prevention regulations. |  |  |  |  |  |
| N19 I worry about lacking knowledge to effectively implement preventive measures. |  |  |  |  |  |
| N20 I am confident in increasing handwashing to prevent the spread of enterovirus and COVID-19. |  |  |  |  |  |
| N21 I am confident in following the proper handwashing steps: palm, back, interlace, cup, thumb, knuckles, wrist. |  |  |  |  |  |
| N22 I am confident I will seek medical attention if I experience symptoms like fever, headache, runny nose, sore throat, cough, diarrhea, or changes in taste or smell. |  |  |  |  |  |
| N23 I am confident I will always wear a mask in crowded public places or on public transport. |  |  |  |  |  |
| N24 I am confident in using diluted bleach or alcohol to disinfect household surfaces like doorknobs and tables. |  |  |  |  |  |
| N25 I am confident I will stay informed on the current situation and prevention policies through news or social media. |  |  |  |  |  |

# Part 4: Reinforcing Factors

Please indicate your level of agreement with the following statements.

|  | Strongly Disagree | Disagree | Neutral | Agree | Strongly Agree |
| --- | --- | --- | --- | --- | --- |
| N26 Parents proactively inform the preschool of their child’s health and absence to assist in prevention. |  |  |  |  |  |
| N27 Parents comply with keeping sick children at home to prevent disease spread. |  |  |  |  |  |
| N28 Parents prepare masks for their children and remind them to wear them during flu season. |  |  |  |  |  |
| N29 Parents take sick children to the doctor and adopt prevention measures. |  |  |  |  |  |
| N30 Preschools monitor educators' health, reminding them of balanced nutrition and sleep for good immunity. |  |  |  |  |  |
| N31 Preschools offer vaccination incentives to encourage higher participation. |  |  |  |  |  |
| N32 Preschools encourage educators to attend disease prevention courses during work hours. |  |  |  |  |  |
| N33 Preschools encourage educators to stay home when sick. |  |  |  |  |  |
| N34 In-service training on health and safety, including disease prevention, helps educators adopt preventive measures. |  |  |  |  |  |
| N35 The Ministry of Health promotes disease prevention through media and encourages preschools to implement it, aiding prevention. |  |  |  |  |  |
| N36 Local education bureaus enforce strict disease control, aiding preschools in prevention efforts. |  |  |  |  |  |

# Part 5: Enabling Factors

Please indicate your level of agreement with the following statements.

|  | Strongly Disagree | Disagree | Neutral | Agree | Strongly Agree |
| --- | --- | --- | --- | --- | --- |
| N37 The preschool provides masks, gloves, and disinfectants for personal protection. |  |  |  |  |  |
| N38 The preschool ensures safe drinking water with regular disinfection and water testing to prevent diseases. |  |  |  |  |  |
| N39 The preschool ensures food safety by maintaining a clean kitchen to prevent bacterial infections. |  |  |  |  |  |
| N40 The preschool uses various methods to remind staff to get flu vaccines. |  |  |  |  |  |
| N41 The preschool provides staff with updates on current infectious diseases. |  |  |  |  |  |
| N42 The preschool installs handwashing stations at entrances and classrooms to promote proper hygiene. |  |  |  |  |  |
| N43 The preschool requires hand disinfection before entry, aiding in preventive measures. |  |  |  |  |  |
| N44 The preschool enforces a sick child policy, helping monitor attendance. |  |  |  |  |  |
| N45 The preschool has a disease contingency plan, aiding response during outbreaks. |  |  |  |  |  |
| N46 The preschool's enterovirus reporting drills help me monitor disease prevention. |  |  |  |  |  |

# Part 6: Prevention Behaviors

Please indicate how often you perform the following behaviors.

|  | Never | Rarely | Sometimes | Often | Always |
| --- | --- | --- | --- | --- | --- |
| N47 I will monitor the current infectious disease situation. |  |  |  |  |  |
| N48 I will provide disease prevention information at preschool. |  |  |  |  |  |
| N49 I will learn and follow disease prevention guidelines. |  |  |  |  |  |
| N50 I will check children upon arrival and ask parents to keep sick children at home. |  |  |  |  |  |
| N51 I will monitor attendance and address sick leave with preventive measures. |  |  |  |  |  |
| N52 I will study disease prevention courses and create health education lessons for children. |  |  |  |  |  |
| N53 I will practice hand hygiene before and after school entry, child interactions, meals, and restroom use. |  |  |  |  |  |
| N54 I will teach children to cover their mouth and nose when sneezing or coughing and dispose of tissues immediately. |  |  |  |  |  |
| N55 I will wear a mask when sick and practice respiratory hygiene, washing hands after blowing my nose. |  |  |  |  |  |
| N56 I will get vaccinated for the flu and COVID-19. |  |  |  |  |  |
| N57 I will maintain a balanced diet, exercise, and get 6-8 hours of sleep to boost immunity. |  |  |  |  |  |
| N58 I will stay home when sick to avoid spreading germs. |  |  |  |  |  |
| N59 I will ensure good ventilation and cleanliness at preschool. |  |  |  |  |  |
| N60 I will learn and follow disease prevention measures. |  |  |  |  |  |
| N61 During flu season, I will share flu-related information in communication books. |  |  |  |  |  |
| N62 I will wear a mask in crowded places. |  |  |  |  |  |
| N63 I will clean frequently touched items like my phone and keyboard with alcohol wipes or diluted bleach. |  |  |  |  |  |
| N64 If I have symptoms like sore throat or fever, I will seek medical attention immediately. |  |  |  |  |  |
